# Temperature modulates PVN pre-sympathetic neurones via transient receptor potential ion channels

**DOI:** 10.3389/fphar.2023.1256924

**Published:** 2023-10-18

**Authors:** Fiona O’Brien, Claire H. Feetham, Caroline A. Staunton, Kathryn Hext, Richard Barrett-Jolley

**Affiliations:** Department of Musculoskeletal Ageing Science, Faculty of Health and Life Sciences, University of Liverpool, Liverpool, United Kingdom

**Keywords:** PVN, PVH, thermoregulation, ion channel, TRPV4, computational model, hypothalamus, patch clamp

## Abstract

The paraventricular nucleus (PVN) of the hypothalamus plays a vital role in maintaining homeostasis and modulates cardiovascular function via autonomic pre-sympathetic neurones. We have previously shown that coupling between transient receptor potential cation channel subfamily V Member 4 (Trpv4) and small-conductance calcium-activated potassium channels (SK) in the PVN facilitate osmosensing, but since TRP channels are also thermosensitive, in this report we investigated the temperature sensitivity of these neurones.

**Methods:** TRP channel mRNA was quantified from mouse PVN with RT-PCR and thermosensitivity of Trpv4-like PVN neuronal ion channels characterised with cell-attached patch-clamp electrophysiology. Following recovery of temperature-sensitive single-channel kinetic schema, we constructed a predictive stochastic mathematical model of these neurones and validated this with electrophysiological recordings of action current frequency.

**Results:** 7 thermosensitive TRP channel genes were found in PVN punches. Trpv4 was the most abundant of these and was identified at the single channel level on PVN neurones. We investigated the thermosensitivity of these Trpv4-like channels; open probability (Po) markedly decreased when temperature was decreased, mediated by a decrease in mean open dwell times. Our neuronal model predicted that PVN spontaneous action current frequency (ACf) would increase as temperature is decreased and in our electrophysiological experiments, we found that ACf from PVN neurones was significantly higher at lower temperatures. The broad-spectrum channel blocker gadolinium (100 µM), was used to block the warm-activated, Ca^2+^-permeable Trpv4 channels. In the presence of gadolinium (100 µM), the temperature effect was largely retained. Using econazole (10 µM), a blocker of Trpm2, we found there were significant increases in overall ACf and the temperature effect was inhibited.

**Conclusion:** Trpv4, the abundantly transcribed thermosensitive TRP channel gene in the PVN appears to contribute to intrinsic thermosensitive properties of PVN neurones. At physiological temperatures (37°C), we observed relatively low ACf primarily due to the activity of Trpm2 channels, whereas at room temperature, where most of the previous characterisation of PVN neuronal activity has been performed, ACf is much higher, and appears to be predominately due to reduced Trpv4 activity. This work gives insight into the fundamental mechanisms by which the body decodes temperature signals and maintains homeostasis.

## Introduction

Prolonged deviation in core body temperature (T_c_) outside a narrow range, results in serious physiological issues incompatible with life, and therefore, it is tightly regulated by a homeostatic system (Morrison and Nakamura, 2011; Gomez, 2014). Changes in environmental temperature produce reflex responses to maintain T_c_ in an optimal range (Kanosue et al., 1991; Kanosue et al., 1994), however, how the brain coordinates such responses is a longstanding and unresolved question.

Early models of temperature regulation were based around the existence of a central integrator comprised of hypothalamic neurones that orchestrate homeostatic responses around a set-point temperature (Hardy, 1961; Hammel and Pierce, 1968). An alternative theory proposes that the brain has no central integrator for T_c_ but instead, various thermoregulatory effectors are thought to be regulated independently, giving the appearance of coordinated action without the existence of a single so-called ‘controller’ (Satinoff, 1978; Romanovsky, 2007; McAllen et al., 2010). Central nervous system (CNS) level control of T_c_ is mediated by a combination of negative feedback and feed-forward mechanisms that share common peripheral thermal sensory inputs (Kanosue et al., 2010; Morrison and Nakamura, 2011). Feedback responses are those that are triggered when T_c_ deviates away from the optimal range, for example, exercise induces an increase in T_c_ by approximately three degrees Celsius (Fuller et al., 1998; Walters et al., 2000). Feed-forward mechanisms on the other hand, are preventative, and are triggered prior to any change in core temperature. The most common feed-forward example is the detection of changes in air temperature (by thermoreceptors in the skin) which trigger thermoregulatory responses that prevent any significant change in T_c_ (Nakamura and Morrison, 2008; Nakamura and Morrison, 2010; Romanovsky, 2014). The hypothalamus contains the primary integrative and rostral efferent components of these circuits, but local thermal stimulation of other areas in the CNS, including several brain-stem neuronal groups and the spinal cord also trigger autonomic thermoeffector responses. Thermosensitive neurones in the preoptic area (POA) of the hypothalamus have been the most studied to date (Kanosue et al., 1991; Kanosue et al., 1994; Kazuyuki et al., 1998; Nagashima et al., 2000; Nakamura and Morrison, 2010; Romanovsky, 2014).

Early experiments showed that stimulation (warming) of the cat (Magoun et al., 1938; Hemingway et al., 1954) and rat (Carlisle and Laudenslager, 1979) POA could trigger dramatic thermoregulatory responses that were similar to those observed by heating the entire animal. Cooling of the POA promotes vasoconstriction, brown adipose tissue (BAT) thermogenesis and shivering in dogs and baboons (Hammel et al., 1960; Gale et al., 1970) and results in baboons signalling for rapid heat reinforcement. Lesioning of the cat POA has been shown to abolish thermoregulatory responses in animals subjected to temperature challenge (Teague and Ranson, 1936; Clark et al., 1939). Direct sensing of changes in skin temperature has been shown to activate POA efferent signals that control thermal effector organs (Morrison et al., 2014; Morrison, 2016). Electrophysiological studies have characterised the intrinsic temperature-sensitive properties of POA neurones in rabbit (Boulant and Hardy, 1974), rat (Hori et al., 1980; Baldino and Geller, 1982), mice (Tan et al., 2016) and dogs (Hardy et al., 1964), however, there are many reports of temperature sensitive neurones outside of the POA (Nakayama and Hardy, 1969; Edinger and Eisenman, 1970; Simon and Iriki, 1970; Wünnenberg and Hardy, 1972; Kobayashi and Murakami, 1982). The neuronal circuitry and projections of the POA are not fully understood but several additional brain regions including the dorsomedial hypothalamus (DMH), the paraventricular nucleus of the hypothalamus (PVN), and the raphe pallidus nucleus have been proposed to act alongside the POA to regulate T_c_ (Morrison et al., 2014; Morrison, 2016; Zhao et al., 2017). The DMH is also recognised as another key player in thermoregulation (Dimicco and Zaretsky, 2007; Morrison and Nakamura, 2011; Heeren and Münzberg, 2013) and stimulation of rat DMH neurones was shown to increase in BAT sympathetic nerve activity (SNA), BAT temperature and Tc (Zaretskaia et al., 2002; Cao et al., 2004; de Menezes et al., 2006).

Several studies using cFos as a marker of activation have shown that mouse PVN neurones respond to both warm and cold ambient temperature change (Bachtell et al., 2003; Bratincsák and Palkovits, 2004). Exposure to a hot environment (39°C) increased cFos expression of rostral ventrolateral medulla (RVLM) –projecting (Cham and Badoer, 2008) and spinally-projecting neurones in the rat PVN (Cham et al., 2006). Anatomical studies using transneuronal viral tracing approaches show that post injection of pseudorabies virus into the rat tail, within the hypothalamic area, the majority of labelled neurones were located in the PVN (Smith et al., 1998). Injection of glutamate in the PVN leads to an increase in BAT temperature in rats and on the other hand, lesioning of the PVN reduced febrile-evoked increases in body temperature, suggesting a role for the PVN in driving sympathetic outflow to BAT, at least in the context of fever (Amir, 1990; Horn et al., 1994; Caldeira et al., 1998; Lu et al., 2001; Leite et al., 2012). Furthermore, more generally, Cabral *et al.* (2012) showed that TRH (a neuropeptide necessary for cold-induced thermogenesis) -neurones in the rat PVN are activated when animals are exposed to short-term cold conditions (Cabral et al., 2012).

Electrophysiological studies have been pivotal to not only characterising the biophysical profile of PVN neurones, but also understanding how the PVN plays a role in the regulation of homeostatic functions. The PVN is typically divided into the parvocellular and magnocellular regions, both with many subdivisions (Paxinos and Watson, 1986; Koutcherov et al., 2000) but in its most simplistic representation, the PVN is divided loosely into the parvocellular area, posterior magnocellular lateral area and the intermediocellular region (dorsal and caudal PVN) where pre-autonomic neurones are most abundant (Kiss et al., 1991; Chen et al., 2014). To date, three distinct electrophysiological phenotypes have been described in PVN neurones (reviewed in Feetham *et al.* (2018)); magnocellular (type I) PVN neurones have phasic bursting patterns and express a rapidly inactivated, or “A-type,” potassium conductance (Tasker and Dudek, 1991; Sonner and Stern, 2007). Parvocellular (type II) PVN neurones express a slowly inactivating delayed rectifier potassium conductance and it is suggested that the differences between types I and II cells may be explained by differential expression of voltage-gated potassium and calcium channels (Luther et al., 2002). Furthermore, in the parvocellular area, there appears to be two different neuronal phenotypes; 1) exhibiting electrophysiological properties similar to neuroendocrine magnocellular cells (Tasker and Dudek, 1993; Stern, 2001) and 2) pre-autonomic/spinally projecting neurones which show a slowly inactivating potassium conductance (Tasker and Dudek, 1993; Barrett-Jolley et al., 2000). In regards to thermosensitivity, Inenaga *et al.* (1987) was the first study to confirm the inherent thermosensitivity of PVN neurones and characterised separate intrinsically “cold-sensitive” and “warm-sensitive” neurones (Inenaga et al., 1987). To our knowledge, this is the only electrophysiological study investigating the temperature sensitivity of PVN neurones, and to date, there has been no molecular characterisation.

The cellular pathways involved in thermo-sensation are well conserved and consist of a set of specialised temperature-gated ion channels that are highly sensitive to a wide temperature range. The thermo-transient receptor potentials (TRPs), a recently discovered family of ion channels activated by temperature, are expressed in primary sensory nerve terminals where they provide information about thermal changes in the environment. There are 4 heat thermo-sensitive TRP ion channels; Trpv1 (activated with temperature >43°C) (Everaerts et al., 2011), Trpv2 (activated with temperature >52°C) (Liu and Qin, 2016), Trpv3 (activated with temperature >32°C) (Peier et al., 2002), TRPM2 (activated with temperature >35°C) (Lamas et al., 2019) and Trpv4 (activated with temperature >27°C) (Güler et al., 2002). In addition, there are 2 identified cold thermo-sensitive TRP ion channels; Trpm8 (activated with temperature <28°C) (McKemy et al., 2002) and Trpa1 (activated with temperature <17°C) (Laursen et al., 2014). PVN ion channels, including those that are thermo-sensitive have recently been summarised in (Feetham et al., 2018).

We have previously shown that Trpv4 is expressed on PVN neurones of CD1 mice (Feetham et al., 2015a; Feetham et al., 2015b). Originally, these channels were considered sensors of cell volume (Liedtke et al., 2000) and in the PVN, we have shown that Trpv4 channels functionally couple to a subtype of Ca^2+^-activated K^+^ channel (SK channel) to sense changes in osmolality, probably mediated by subtle changes in cellular volume (Feetham et al., 2015b). We also found that ICV injection of hypotonic artificial cerebrospinal fluid (ACSF) into CD1 mice decreased mean blood pressure, but not heart rate and this effect was abolished by treatment with the Trpv4 inhibitor RN1734 (Feetham et al., 2015a). In another recent study, we found that systemic administration of the highly selective lipid-soluble Trpv4 antagonist GSK2193874 resulted in tail blood-flow dynamics that were in-compatible with a local (vascular smooth muscle or endothelial cell) mechanism (O'Brien et al., 2022). In light of the data reported here, we hypothesised that PVN Trpv4 ion channels also play a role in thermoregulation.

Many studies have shown that Trpv4 can be activated by heat >27°C (Güler et al., 2002; Watanabe et al., 2002; Clapham, 2003) as well as mechanical stimuli. Trpv4 immunoreactivity is present in a number of brain regions known for producing thermoeffector responses including the POA (Güler et al., 2002), the PVN (Feetham et al., 2018; Shenton and Pyner, 2018), but also in the vasculature where its activation produces vasodilation (Filosa et al., 2013).

Therefore, the aim of this study was to characterise the thermosensitivity of a subpopulation of PVN neurones; we have already shown that Trpv4 channels are present in the PVN but here, we used RT-PCR to identify additional thermosensitive targets. We pharmacologically identified Trpv4-like channels from PVN neurones and characterised their intrinsic thermosensitive properties at the single-channel level. We built on our previous mathematical model to predict that neuronal activity should increase as temperature is decreased; we validate this model with recordings from PVN neurones. We show that the temperature-sensing capabilities of PVN neurones is complex, and is likely to involve multiple ion channels, including Trpv4, and another known thermosensitive ion channel, Trpm2.

## Methods

### Animals

CD1 mice were housed at 22°C–24°C in a 12 h light/dark cycle-controlled facility with *ad libitum* access to food and water. Animals were sacrificed by UK Home Office approved “Schedule 1” methods (see details below) for all *in-vitro* work and all experiments were approved by the Home Office, UK. We present data from a total of 31 mice. For ethical reasons we used male mice so that the females could be reserved for breeding programs to reduce over-all animal usage within the facility.

### Quantitative PCR

Young adult (6–8 months) and old (24 months) mice were killed by Schedule 1 methods (cervical dislocation followed by exsanguination) and the hypothalamic area was blocked and transversely sliced (600 μM thickness). The PVN was identified using the third ventricle and fornix as markers and was punched out using a 1.5 mm biopsy punch. PVN punch biopsies were suspended RNA-free water and homogenised by passing the lysate through 20-gauge needle multiple times. To obtain the required volume of mRNA, samples were pooled, with between 2-3 PVN punch biopsies pooled together. RNA extraction was carried out using the RNeasy Plus Micro kit, together with gDNA eliminator and MinElute spin columns (Qiagen, UK) and analysed for concentration, purity and quality using the NanoDrop™ 2000 (Thermo Scientific, UK). cDNA synthesis (mRNA) was performed using the RT2 First Strand kit (Qiagen, NL) according to the manufacturer’s protocol. Using the Neuronal ion channel plate (Qiagen, UK), 84 ion channels as well as housekeepers were measured in each sample. qPCR analysis was performed using the Stratagene MX3000P RT-PCR System (Stratagene, La Jolla, CA) in a 25-μL reaction mixture. Expression relative to mean of 3 housekeeper genes (Actb, Ldha, Rplp1) cycle thresholds (Ct) for that sample is presented as the ΔCt.

### Brain slice preparation

CD1 mice aged 2–3 weeks were killed by Schedule 1 methods (cervical dislocation and exsanguination) and the brain was swiftly removed and placed in ice-cold low Na^+^/high-sucrose artificial cerebrospinal fluid (ACSF) and sliced as previously described (Feetham et al., 2015b). In brief, coronal PVN slices were prepared using a Leica VT1000S and stored in a multiwell dish containing physiological ACSF. Slices were kept at 35°C–37°C with continuous perfusion of 95% O_2_/5% CO_2_ and left to recover for at least 1 h before recording.

### Electrophysiology

Thick-walled patch-pipettes were fabricated using fire-polished 1.5 mm o. d. borosilicate glass capillary tubes (Sutter Instrument, Novato CA, United States ) using a two-step electrode puller (Narishige, Japan), final resistance when filled 5–8 MΩ. Neurones were visualised using a Hitachi KP-M3E/K CCD camera attached to a Nikon Eclipse microscope with an effective magnification of ∼1,000x. Cell-attached patch clamp electrophysiology was performed as previously described using an Axopatch 200b amplifier (Molecular Devices Axon Instruments, United States ) (Feetham et al., 2015b). Theory and justification of action current measurement is given by Fenwick et al. (1982). For spontaneous action current recordings, analogue data were further amplified with a Tektronix FM122 (Beaverton, OR, United States ) AC-coupled amplifier. The temperature of the recording bath was maintained using the npi electronic TC-10 (Scientifica, UK). In all cases, data were low-pass filtered at 1 kHz and digitised at 5 kHz with a Digi Data 1200B interface. Recording solutions are described below and junction potentials were calculated using JpCalc (Barry and Lynch, 1991). Slices were allowed to equilibrate for 15 min in the recording chamber prior to recording. Temperature was controlled using a commercial feedback temperature control unit (Thermoclamp, AutoMate Science, Berkeley, California, United States), with thermocouple placed within the recording chamber alongside the PVN slice. Temperature was changed in 5°C increments.

### Analysis of electrophysiological recordings

Single channel recordings were digitally filtered at 1 kHz in WinEDR (University of Strathclyde, UK). Open and closed levels were assessed by all-points amplitude histograms and were used to create current-voltage IV) curves. Single channel events were idealized using the segmental K means (SKM) methods (Qin, 2004) using QuB software (SUNY, Buffalo, NY) and open probability (*Po*) was determined from the idealised record as previously described (Lewis et al., 2013). For dwell time analysis, dead-time was set to three sample intervals (0.3 ms) and recordings where only a single channel was gating were used. Open and closed dwell times were log binned according to the methods of (Sigworth and Sine, 1987) and fitted with an exponential log probability density function (pdf) in Clampfit 10.3 (Molecular Devices, Sunnydale California). The number of time constants for each distribution was determined using a log-likelihood ratio test in Clampfit 10.3 at a confidence level of *p* = 0.95.

Individual sets of model kinetic rates were obtained by fitting the idealised data using the MIL algorithm implemented in QuB. Missed events during maximum interval likelihood (MIL) rate optimisation were automatically accounted for in QuB by computation of a corrected Q matrix in the MIL algorithm.

Analysis of action current frequency was performed using WinEDR for acquisition of data and then a custom program designed to detect action currents based on an adaptive threshold routine.

### Solutions

Cell-attached patch recordings were made using the following solutions: ACSF composition (mM): 127 NaCl, 1.8 KCl, 1.2 KH_2_PO4, 2.4 CaCl_2_, 1.3 MgSO_4_, 26 NaHCO_3_ and 5 glucose. Pipette solution for action current and single channel recordings composition (mM): 35 KG; 5 KCl; 100 NaCl and 10 HEPES (pH 7.4) with NaOH. All experiments were performed in the daytime (11:00–17:00 h) to limit the effects of circadian rhythm on activity of the cells used (Belle et al., 2009).

### Design of the computer model

Mathematical models were constructed in Python using open-source NEURON libraries (Hines and Carnevale, 1997; Hines et al., 2009). Our model was based on that of Feetham et al. (2015b) (Feetham et al., 2015b); in brief, inputs arise from both excitatory ‘Netstim’ neurones and inhibitory interneurones, see Figure 5A. The interneurones are also driven by excitatory ‘Netstim’ neurones. Since computer power has increased significantly, the model has been updated in several ways: a) The new model uses stochastic channels rather than “density” (deterministic equations), since the noise added by stochastic simulation allows for more authentic simulation (Cannon et al., 2010). To obtain single channel rate constants for Kv channels we fitted our whole-cell Kv data using a Monte Carlo bootstrap approach within Python. Netstim activities are also now scattered stochastically around the fixed means previously used by Feetham et al. (2015b) (Feetham et al., 2015b). b) We replaced osmotic sensitivity of Trpv4 with a temperature-sensitive channels, using our experimentally measured rate constants, stochastic model and conductance, see (Figure 3A). Temperature dependence was included by applying Q10 to each of the forward rate constants. We also added stochastic SK channels from the model of Moczydlowski and Latorre (Moczydlowski and Latorre, 1983) and a hypothetical TRPM2-like channel using arbitrary base rate-constants, but Q10 (15.6) measured by Togashi et al. (2006). Ion channel permeabilities were from Alexander et al. (2015). c) We replaced the former bulk Ca^2+^ accumulation mechanism (Feetham et al., 2015b) with a new reaction diffusion (RXD) model (McDougal et al., 2013) including central Ca^2+^ ion buffering. All code will be made freely available on GitHub, and if possible, ModelDB.

### Data analysis

All data on graphs are shown as mean ± SEM. Simple comparisons were made using a two-tailed Student’s paired *t*-test. Unless otherwise stated, multiple comparisons were made using a repeated measures ANOVA with multiple comparisons by Tukey’s *post hoc* test or against control levels using Dunnett’s *post hoc* test where appropriate. A value of *p* < 0.05 was taken as significant.

### Materials

GSK2193874 (80 nM, included in the patch pipette), gadolinium (100 μM) and Econozole (10 μM) were applied to the perfusion solution where stated. All drugs were purchased from Sigma-Aldrich and were all dissolved in DMSO and diluted to a final working concentration of no more than 0.01% DMSO (0.01% DMSO had no effect alone).

## Results

### Gene expression levels of thermosensitive TRP channels in punches of mouse PVN

**FIGURE 1:**
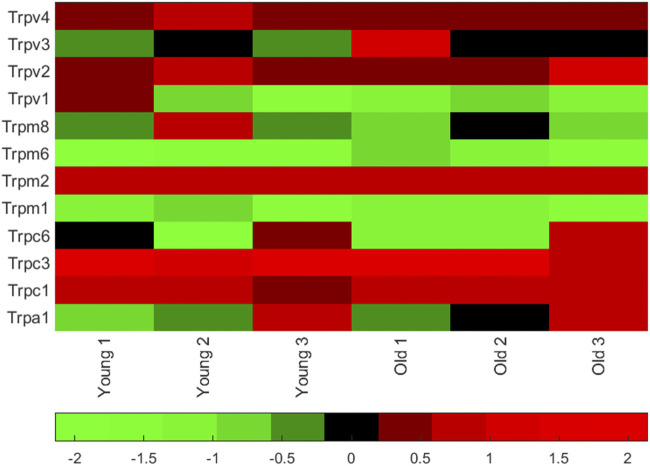
Thermosensitive TRP channel gene expression in PVN punches from young mice. Matlab heatmap standardized (by column) difference ((delta)) in cycle threshold (ΔCt) levels for seven known TRP channel mRNA in punches of the PVN (from 6 animals, 3 young adult and 3 old adults). Red genes are relatively highly expressed and the green are low expression. Mean ΔCt was lowest (highest mRNA abundance) for Trpv4 (see values in the text). A full dataset of 84 ion channel mRNA ΔCt levels measured in younger (6–8 months) and older (26 months) mice are given together with heatmaps in the Supporting Material.

We measured mRNA (by quantitative qPCR) of the warm activated Tprv1 (ΔCt 11.50 ± 2.97, n = 3), Tprv2 (ΔCt 5.29 ± 0.32, n = 3), Tprv3 (ΔCt 9.39 ± 0.35, n = 3), Tprv4 (ΔCt 4.46 ± 0.22, n = 3) and Trpm2 (ΔCt 4.04 ± 0.36, n = 3) channels and mRNA levels of the cold activated Trpm8 (ΔCt 8.78 ± 1.50, n = 3) and Trpa1 (ΔCt 8.48 ± 2.34, n = 3) channels (Klein et al., 2015; Nazıroğlu and Braidy, 2017). Note lower ΔCt means higher mRNA abundance. Therefore, in young mice (the age used in the rest of this work) Trpv4 and Trpm2 were the most abundantly expressed of these thermosensitive TRP channels (Figure 1), with Trpv2 also being highly expressed across age groups. Full datasets for a set of 84 ion channel genes including these, in both young adult and old mice are included in the Supporting Material.

### Identification of Trpv4 channels on PVN neurones

**FIGURE 2:**
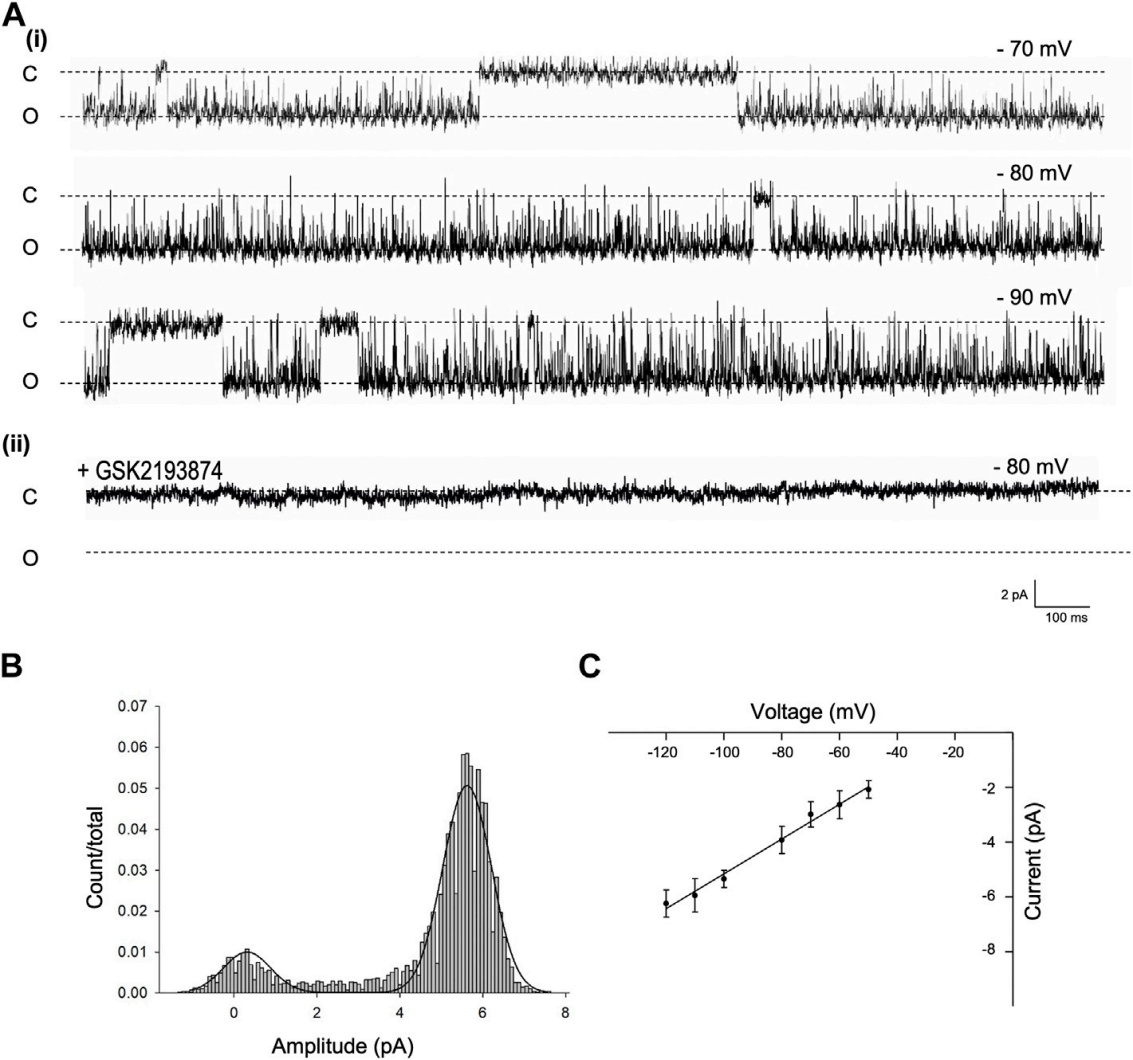
Single channel properties of Trpv4-like channels from PVN neurones **(A).** Representative single-channel current fluctuations through Trpv4-like channels from mouse PVN neurones. Holding potentials are indicated on the trace. The open and closed channel levels are indicated by O and C, respectively. This trace is representative of 10 experiments where Trpv4-like channels were observed and these currents were absent in the presence of the Trpv4 inhibitor GSK2193874. **(B)**. The amplitude histogram is shown for ion channels shown A, at Vm −70 mV. **(C)**. Current-voltage relationship for Trpv4-like channels. Mean ± SEM is shown (n = 9).

To identify and characterise the single-channel gating of PVN Trpv4 channels, we used cell attached electrophysiology on PVN neurones. In control conditions, a Trpv4-like channel was identified in 62% of useable recordings with a conductance of 59.7 ± 1 pS and Vrev of −18.89 mV (95% CI: −26 to −10 mV, at 22°C) (Figure 2Ai, n = 10). This channel was absent when cells were patched in the presence of the specific Trpv4 antagonist GSK2193874 (80 nM, Figure 2Bii)).

### PVN Trpv4-like channels are sensitive to temperature

**FIGURE 3:**
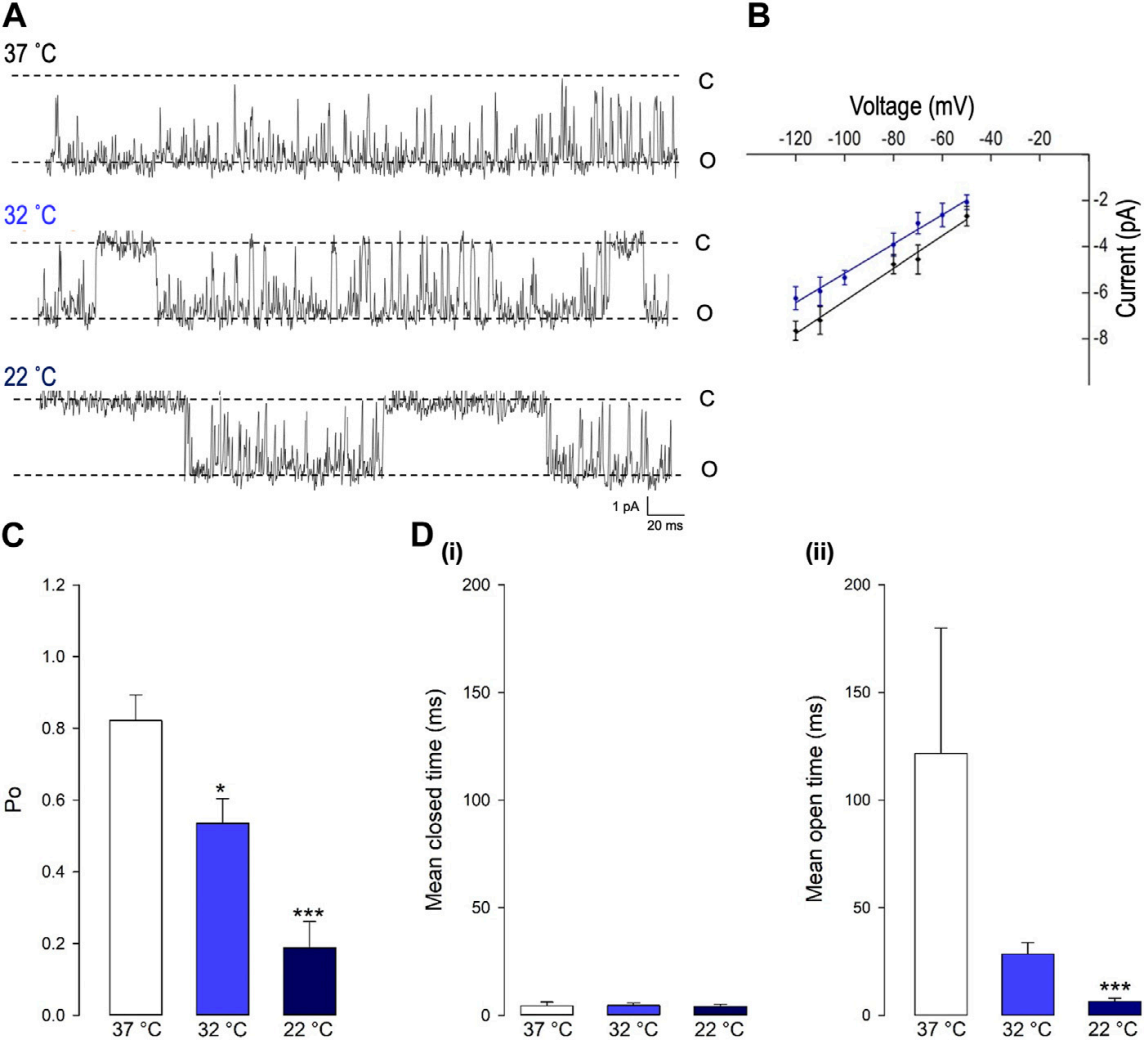
The gating of PVN Trpv4-like channels is temperature sensitive. **(A)** Representative single-channel current fluctuations through Trpv4-like channels from mouse PVN neurones at 37°C, 32°C and 22°C. The open and closed channel levels are indicated by O and C, respectively. **(B).** Current-voltage relationship for Trpv4-like channels at 37°C (red circles) and 22°C (blue circles). **(C).** The *Po* of Trpv4-like channels at 37°C, 32°C and 22°C is shown. **(D).** Mean open and closed times for Trpv4-like channels. Mean ± SEM is shown (n = 7 at 22°C, n = 7 at 32°C and n = 9 at 37°C).

Decreasing temperature dramatically reduced the open probability (*Po*) of this Trpv4-like channel (*p < 0.05, ***p < 0.001, Figure 2A and B, n = 9). As shown in Figure 3A, at 37°C, Trpv4-like channels were predominately open with only brief closing events and at lower temperatures, there was an apparent reduction in open durations. We found that the decrease in mean *Po* was mediated by profound decrease in the mean open time (***p < 0.001, Fig. 3D, n = 9) with no change in mean closed time. We also observed a small but significant decrease in Trpv4-like channel conductance when temperature was decreased (Figure 3B, n = 9, blue line).

**FIGURE 4:**
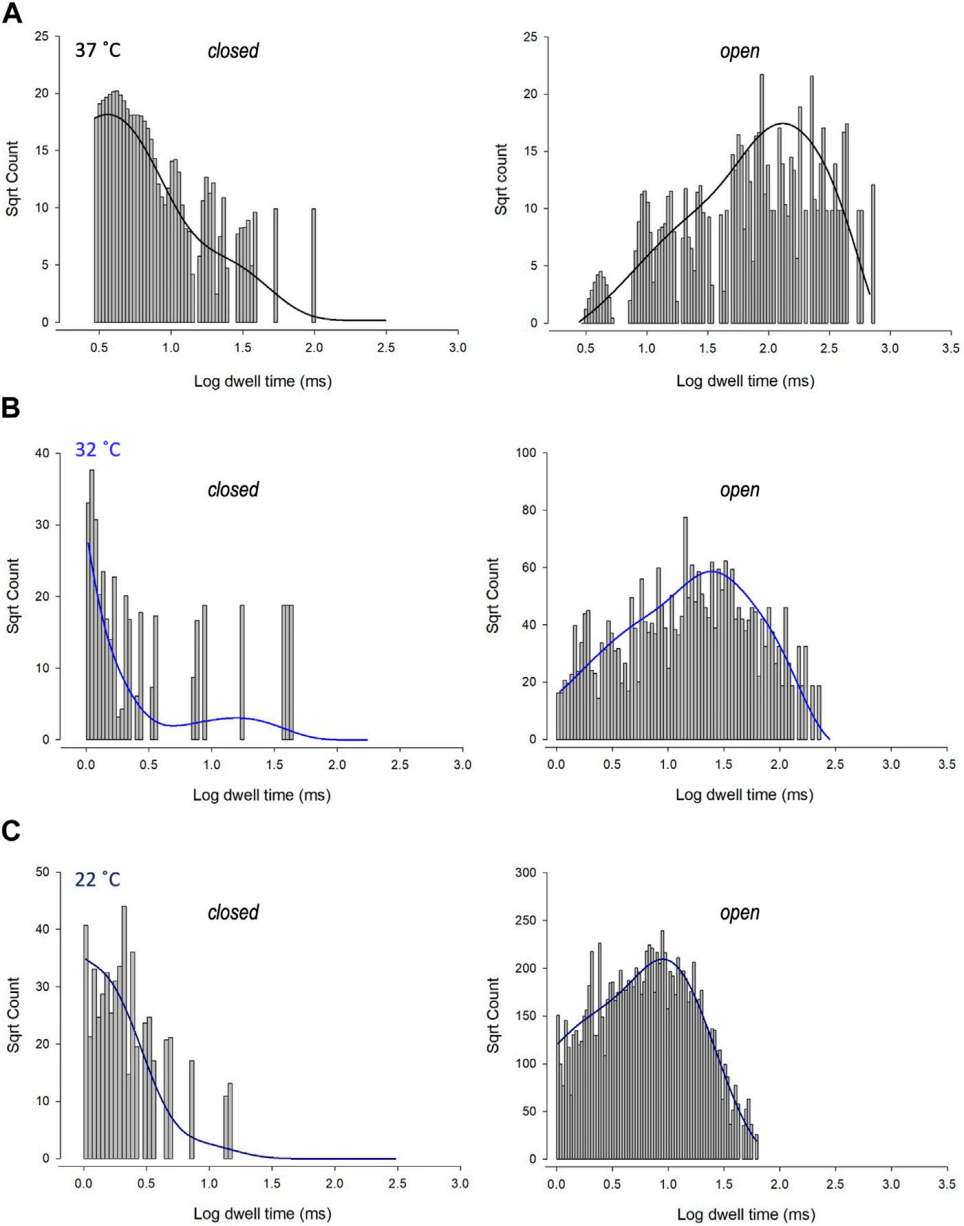
Kinetics of Trpv4-like channels from PVN neurones. Kinetic analysis of Trpv4-like channel dwell-times from PVN neurones recorded in cell attached-patch mode at 37°C **(A)**, 32°C **(B)** and 22°C **(C)**. Closed (left) and open (right) dwell-times were fitted with 3 exponentials (solid lines). Data are transformed with log-binning (*x*-axis) and square root of frequency (*y*-axis) so that exponential time constants are visible as peaks (Sigworth and Sine, 1987). Mean values are given in Table 1 and a kinetic schema in Figure 5.

To investigate changes in gating further, we performed detailed analysis of the open and closed dwell-time distributions. Data were fit with 3 open and closed states. A representative example is shown in Figure 4. Lower temperatures markedly reduced 2 out of 3 open *taus* (*tau*_O2_ and *tau*_O3_), with no significant change in any of the closed *taus* (Table 1). Thus, the mechanism for the temperature evoked decrease in Trpv4-like *Po* observed when cooled is a decrease in mean open dwell times.

**TABLE 1:** Time constants and percentage areas are shown as obtained from maximum likelihood fitting of pdfs to closed and open lifetime distributions of Trpv4-like channels from PVN neurones at 22°C, 32°C, and 37°C. Data are presented as mean ± SEM for 7-9 experiments (*p < 0.05, **p < 0.01, ***p < 0.0001).

Closed state dwell times					
22°C		32°C		37°C	
tau (ms)	Area (%)	tau (ms)	Area (%)	tau (ms)	Area (%)
0.58 ± 0.01	58	0.75 ± 0.02	65	1.24 ± 0.3	69
5.26 ± 0.02	30	5.87 ± 0.2	27	7.28 ± 2.3	24
71.22 ± 3.9	12	49.96 ± 6.9	7	82.19±	7

Open state dwell times					
22°C		32°C		37°C	
tau (ms)	Area (%)	tau (ms)	Area (%)	tau (ms)	Area (%)
1.32 ± 0.68	40	1.88 ± 0.41	38	2.73 ± 0.32	28
3.99 ± 1.65	39	14.14 ± 2.3**	39	40.07 ± 8.22***	49
41.51 ± 12.06	21	106.55 ± 19.36*	23	224.57 ± 48.17**	23

### *In silico* analysis of Trpv4 inhibition and prediction of PVN action current frequency

**FIGURE 5:**
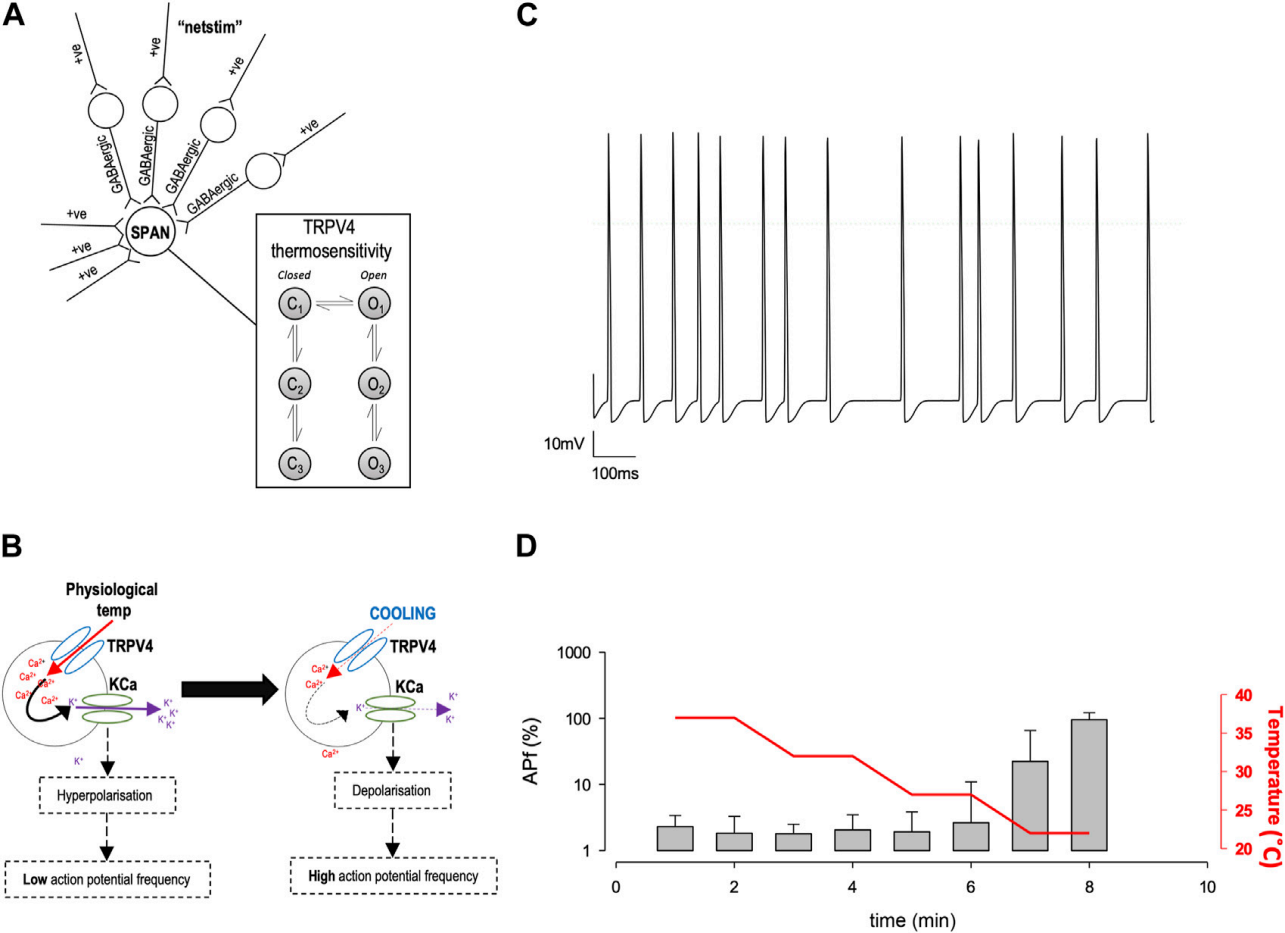
*In silico* model of PVN neurones. **(A)** The simple scheme adapted from (Feetham et al., 2015b) whereby influx of Ca^2+^ increases K_Ca_ channel activity which hyperpolarizes the cell and increases the inward flux of Ca^2+^, by increasing the driving force for Ca^2+^ entry. **(B).** A computer model was adapted from (Feetham et al., 2015b) in NEURON, which includes thermosensitive TRP channels and allows an accumulation of Ca^2+^ into the cell, which is linked to a K_Ca_ channel. Within the model, we can change temperature and simulate action currents, shown in **(C). (D).** Increase in action potential frequency when temperature is decreased (n = 5 simulation runs, mean and SD of the 5 runs shown in **(D)**.

Characterisation of precise single ion channel gating facilitates the computation of neuronal action potential firing properties, which may correlate to how sympathetic output may be controlled. Our working hypothesis of PVN neurones is that as temperature decreases, decreasing activity of calcium permeable TRP channels leads to a decrease in the activity of nearby Ca^2+^-activated potassium channels (K_Ca_), respectively, without causing biologically significant changes in global [Ca^2+^] (Feetham et al., 2015b). We therefore hypothesized that the reduction in Trpv4 activity observed at cooler temperatures would result in an increase in the frequency of spontaneous action currents (ACf) from PVN neurones (Figure 5B). To test the plausibility of this hypothesis quanitatively, we constructed a mathematical model of a PVN neurone cell based on our previous model (Feetham et al., 2015b), as shown in Figure 5A. Our stochastic model predicted that decreasing temperature from 37°C to 22°C would increase the ACf from PVN neurones (*p < 0.0001, n = 5, Figures 5C,D).

### PVN neuronal action current frequency is increased at low temperatures

**FIGURE 6:**
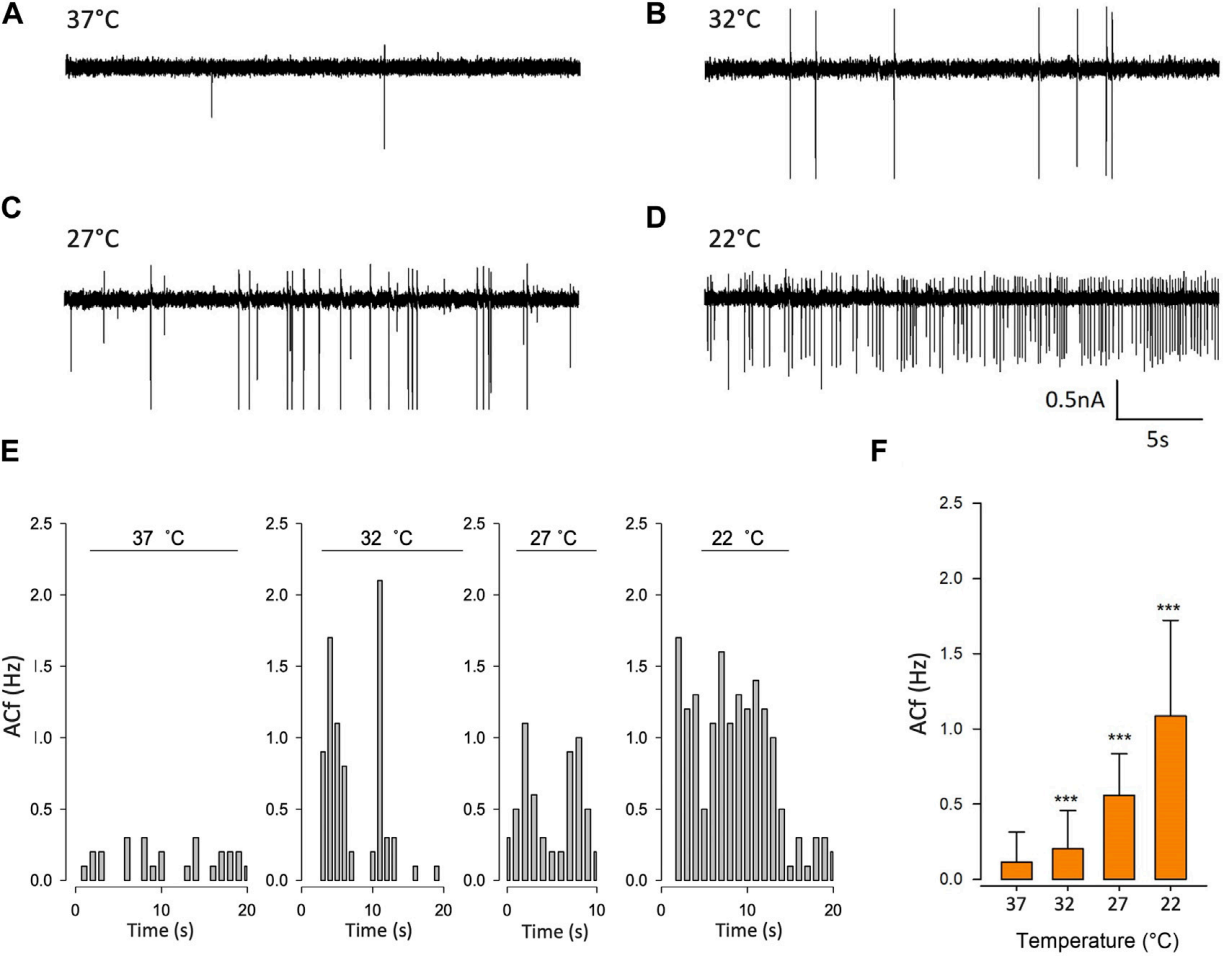
Temperature decreases action current frequency of PVN neurones. **(A).** Representative spontaneous firing of action currents from PVN neurones are shown at physiological temperature (37°C) and at lower temperatures of 32°C **(B)**, 27°C **(C)** and 22°C **(D)**. **(E).** Representative frequency histogram showing action current response of a PVN neurone to decreasing temperature. **(F).** The mean temperature responses are shown for PVN neurones. Data is presented as mean ± SEM (n = 6, ***p < 0.001, Friedman Test).

To validate our mathematical model, we used cell-attached patch-clamp electrophysiology on PVN neurones. Neurones were patched in the PVN using cell attached-patch methods and ACf recording; we selected neurones on the basis that they were firing action currents at rest. We found that ACf was significantly higher at lower temperatures (***p < 0.001, Figure 6, n = 7). The maximum effect was observed at room temperature (22°C) where there was a 10-fold increase in ACf (***p < 0.001, Figure 6F, n = 7). It is worth nothing that 27°C (the temperature threshold for Trpv4 activation), is where we observed the largest increase in ACf compared to control (**p < 0.01, Figure 6C, n = 6).

### Temperature sensitivity of PVN neurones

**FIGURE 7:**
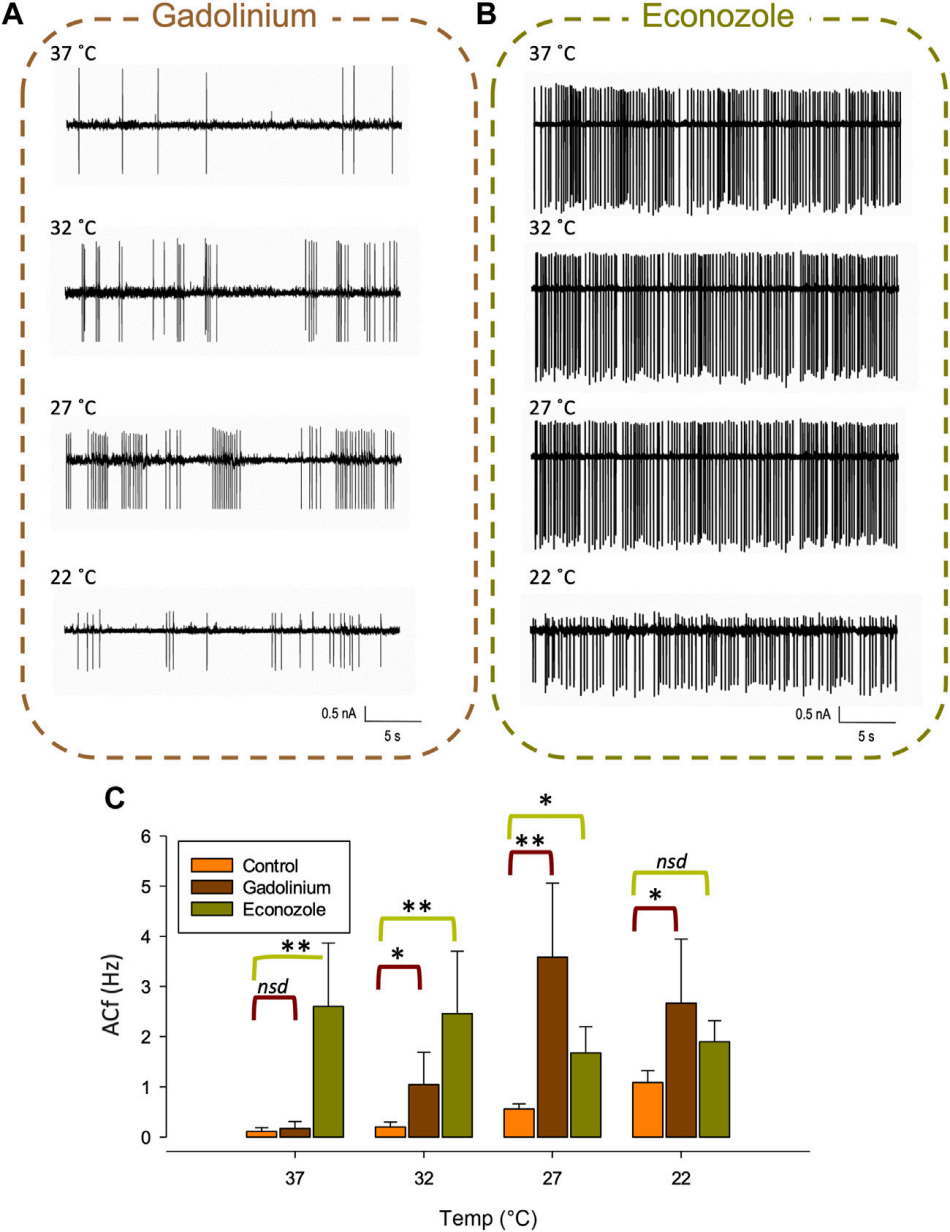
Pharmacological inhibition of various TRP channels on temperature sensitivity of PVN neurones. Representative spontaneous firing of action currents from PVN neurones are shown at physiological temperature (37°C) and at lower temperatures of 32°C, 27°C and 22°C in the presence of bath applied **(A)** gadolinium or **(B)** econazole. In **(C)**, the mean temperature responses are shown for PVN neurones in the presence of the non-specific inhibitor gadolinium, 100 µM (brown) or the Trpm2 blocker econazole, 10 µM (green). Data is presented as mean ± SEM (n = 6 for control, n = 4 for gadolinium, n = 5 for econazole, *p < 0.05, **p < 0.01, Friedman Test).

At lower recording temperatures, we observed an increase in ACf which is likely mediated by a decrease in K_Ca_ activity. In this temperature range, a decrease in activity of *any* of our identified warm-activated Ca^2+^-permeable TRP channels (Trpv4, Trpv3 and Trpm2) could account for this phenomenon (see Figure 1B). We therefore repeated our temperature protocol in the presence of gadolinium (100 µM) which inhibits both Trpv4 (Liedtke et al., 2000) and Trpv3 (Tousova et al., 2005) and found that while the temperature response persisted from 37°C to 27°C (**p < 0.01, Figure 7C, n = 6) it did not increase further as the temperature was lowered to 22°C.

We also recorded ACf at different temperatures in the presence of econazole (10 µM) which is known to block the thermosensitive Trpm2 channel (Hill et al., 2004). We found that ACf no longer changed with recording temperature, indicating that the temperature response was inhibited, (Figure 7B, n = 5). However, at the higher temperatures of 37°C and 32°C, ACf was markedly higher to that recorded in control conditions (no drug) (**p < 0.01, Figure 7C, n = 5).

## Discussion

In this study, we identify thermosensitive TRP channels in the PVN using RT-PCR, of which, Trpv4 is one of the most abundantly expressed. We characterised the single-channel properties of pharmacologically identified Trpv4-like channels on PVN neurones. We report that these channels are thermosensitive, with decreased activity at lower temperatures, and although our mathematical model predicts that our single channel results could account for the increase in neuronal PVN activity we observed at lower temperatures, we find that the temperature sensitivity of PVN neurones is complex and is likely mediated by the cooperated orchestration of multiple thermosensitive channels, including Trpv4 and Trpm2. Our experiments detected the mRNA of 7 TRP channel genes in total in mouse PVN punches. Whilst Trpv4 and Trpm2 were highly expressed with ΔCt of approximately 4, others were present at lower expression; Trpm2≥Trpv4 > Trpv2 >> Trpa1≥Trpm8 > Trpv3 >> Trpv1. With ΔCt of over 11, there may be a question as to whether the lowest of these, Trpv1, was genuinely expressed in mouse PVN. Whilst it was reported to be present in *rat* PVN Trpv1, specifically in autonomic spinally projecting neurones (Li et al., 2004), elsewhere it was reported to be *absent* from adult mouse PVN using *in situ* hybridisation (Cavanaugh et al., 2011). One possibility is that the mouse punches may have clipped the neighboring DMH, and other would be simply that transcription levels are low and variable.

**FIGURE 8:**
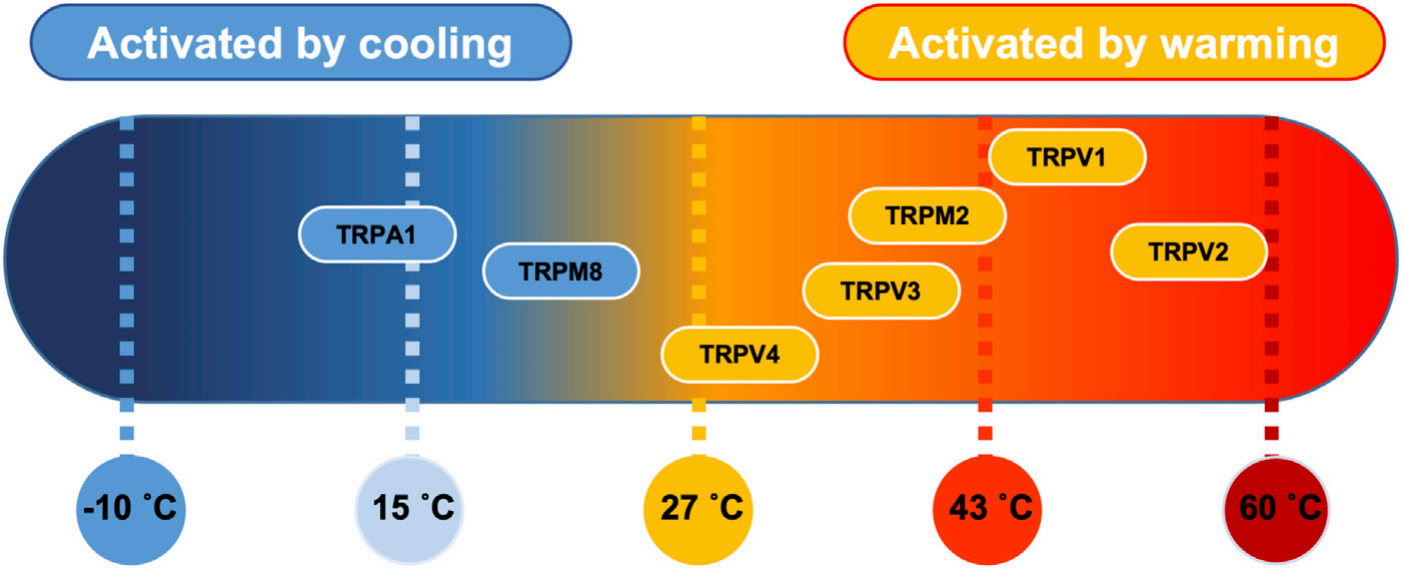
Thermosensitive TRP channel genes. Established distribution of transient receptor potential (TRP) channels in PVN tissue as a function of their temperature threshold. TRP channels may be activated by increases in temperature (orange) or by lowering the temperature (blue) (Klein et al., 2015). Image modified under BY4.0 Creative Commons Licence from (Lamas et al., 2019).

Several TRP channels are temperature sensitive (Figure 8) and our results here confirm that Trpv4-like channels are present on PVN neurones; we have previously illustrated the expression profile of Trpv4 within the PVN using immunohistochemistry (Feetham et al., 2015b) and have shown that application of the selective Trpv4 *agonist* GSK1016790A decreased the firing rate of PVN neurones (Feetham et al., 2015b). At the whole animal level, we have shown that ICV injection of the Trpv4 inhibitor RN1734 prevents the effect of hypotonic ASCF on blood pressure (Feetham et al., 2015a) and centrally located Trpv4 channels may be responsible for the vasodilatory effect of systemic injection of a Trpv4 agonist (O'Brien et al., 2022).

In this paper, we characterize the biophysical properties of Trpv4-like channels from PVN neurones using patch-clamp electrophysiology. A Trpv4-like channel was pharmacologically identified with conductance and reversal potential similar to those reported for recombinant Trpv4 channels (Watanabe et al., 2002).

Our results are in agreement with several other studies reporting Trpv4 expression in the PVN; Carreño et al. (2009) show Trpv4-positive cells colocalised with vasopressin (AVP) in both the magnocellular and parvocellular rat PVN (Carreno et al., 2009). However, Shenton & Pyner (2018) reported that Trpv4-immunopositive spinally projecting (pre-autonomic) neurone cell bodies were rare in the rat PVN, with immunoreactivity predominately within the magnocellular area (Shenton and Pyner, 2018). This is rather in contrast to our mouse data, both previously (Feetham et al., 2015b) and in this paper where we find Trpv4-like channel activity in 62% (10/16) of our anatomically and morphologically defined neurones in the parvocellular PVN area. That said, there are major differences between the mouse and rat PVN, for example, unlike the rat PVN, the mouse PVN is not well differentiated and magnocellular and parvocellular neurones are often indistinguishable (Biag et al., 2012). It would now be fascinating to see co-staining of Trpv4 and retrogradely labelled *mouse* PVN too since such data can identify spinally projecting (pre-autonomic) neurones directly. In addition, there have been reported mechanistic species differences too, for example, acute leptin injection induced significant pSTAT3 (a marker of leptin-responsive cells) expression in the rat PVN but not in the mouse. They also reported that the rat PVN exhibited a denser proopiomelanocortin (POMC) innervation compared to the mouse (Campos et al., 2020). In addition, major differences in neuronal populations in different areas of the brain have been reported between species, for example, numerous CRF-ir neurones in the medial preoptic area of rats were barely observed in mice and numerous CRF-ir neurones in the dorsal nucleus of vagus nerve (DMN) of mice that were not present in rats (Wang et al., 2011). In addition, Trpv4 channels have been shown to translocate; Baratchi *et al.* (2016) showed that in human umbilical vein endothelial cells (HUVECs) and in human embryonic kidney 293 cells (HEK293) transfected with Trpv4, sheer stress triggered translocation of Trpv4 to the plasma membrane within seconds of treatment (Baratchi et al., 2016). Again, in a later publication, they demonstrated that in HUVECs, upon application of shear stress, clusters of Trpv4 channels dispersed into individual channels and translocated from the basolateral to the basal membrane (Baratchi et al., 2017). It is therefore plausible that any shear stress or mechanical perturbation may cause additional Trpv4 channels to be translocated to the plasma membrane and be observed more easily at the single channel level.

We find that the gating of Trpv4-like channels is profoundly affected by temperature; *Po* decreased when the temperature was lowered, mediated by a decrease in mean open durations. Trpv4-like channels on PVN neurones were almost maximally activated at normal physiological body temperature, which has been reported elsewhere for TRPV4 channels expressed in HEK293 cells (Watanabe et al., 2002) and for Trpv4 in isolated hippocampal pyramidal neurones (Shibasaki et al., 2007). The general consensus is that at physiological temperatures, Trpv4 channels may serve as constitutively open Ca^2+^ entry channels that are sensitive to small deviations in temperature (Watanabe et al., 2002) and control neuronal excitability *in vitro* and *in vivo* (Shibasaki et al., 2007; Shibasaki et al., 2015).

Although the molecular dynamics behind heat activation of Trpv4-like channels in the PVN is not known, Watanabe et al. (2002) illustrated that whilst 4αPDD can activate Trpv4 channels in both the cell-attached and cell-free patch clamp configurations, heat application could *only* activate channels in the cell-attached mode, suggesting that there might be an intrinsic heat sensitive ligand or messenger that can active Trpv4 channels from the inside rather than heat activating the channel directly (Watanabe et al., 2002). We do not know the mechanism behind the heat activation of Trpv4 channels on PVN neurones as we only patched in the cell-attached mode but we could hypothesise that there may be a similar mechanism here. Furthermore, given the limitations of our own study here, it is possible that rather than including a specific temperature sensible domain, the channel could be responding to changes in temperature via changes in membrane fluidity that increases with temperature, as proposed for other ion channels many years ago (Romey et al., 1980).

We have previously shown that pharmacological activation of Trpv4 decreases spontaneous ACf of PVN neurones, mediated by the Ca^2+^-induced (Romey et al., 1980) activation of K_Ca_ channels (Feetham et al., 2015b). Activation of K_Ca_ channels induces hyperpolarisation, which in turn, draws greater Ca^2+^ into the cell by increasing the driving force for Ca^2+^ entry, setting up a positive feedback loop (Guéguinou et al., 2014; Feetham et al., 2015b). In our paper and in others, our mathematical model showed that even in the absence of large global changes in Ca^2+^, Trpv4 could permit entry of sufficient Ca^2+^ to activate local SK channels, due to a combinational of local Ca^2+^ signaling domains that limit the diffusion of Ca^2+^ ions after they have entered the cell and the close proximity that often exists between the Ca^2+^ permeable channel (Trpv4 channel) and the Ca^2+^ signaling system (K_Ca_ channel) (Neher, 1998; Augustine et al., 2003; Fakler and Adelman, 2008).

We have made fundamental changes to our mathematical model. Firstly, we added probabilistic or stochastic gating of ion channels (gating between open and closed states) which attributes to ‘channel noise’ in neuronal activity (White et al., 2000; Goldwyn and Shea-Brown, 2011). It has been demonstrated that the Hodgkin-Huxley derived neuronal models with discrete Markovian ion channel kinetics instead of the usual rate equations can lead to spontaneous generation of action potentials (Lecar and Nossal, 1971; Skaugen and Walløe, 1979; Strassberg and DeFelice, 1993; Hänggi, 2002). In addition, including stochastic behaviour of ion channel gating imparts neuronal noise (Cannon et al., 2010) that has been shown to effect the variability of spike timing (Schneidman et al., 1998), firing coherence (Sun et al., 2011) and the regularity of spontaneous spike activity (Ozer et al., 2009). We therefore employed stochastic channels where possible, using the established architecture in NEURON (Hines and Carnevale, 1997). Also, in the previous model we used intracellular Ca^2+^ buffering that was available in NEURON (Hines and Carnevale, 1997) but here, we updated to the newer reaction diffusion (RXD) meshwork within pyNeuron (McDougal et al., 2013; Newton et al., 2018). This allowed us to model very local changes of Ca^2+^ ions in the direct region of the TRP channel-Ca^2+^-activated potassium channel microdomain. This microdomain approach is critical for understanding functional couplings. Clearly Ca^2+^ concentration does not need to increase across the entire body of the cell, and indeed this is an observation that has been verified in other cell types (Fakler and Adelman, 2008).

Our mathematic simulations predicted that the reduction in Trpv4 *Po* observed at lower temperatures would decrease ACf if the same Trpv4/SK mechanism was at play. In our electrophysiology experiments on neurones in the parvocellular PVN area, we observed a higher ACf at lower temperatures (Figure 6). At 37°C, these neurones have little spontaneous activity Figure 6C), which was 1.8-fold higher even when temperature was only 5°C cooler. Our previous work (Feetham et al., 2015b) was performed at room temperature and therefore in this study, we included bath temperatures as low as 22°C for comparative reasons.

In the presence of gadolinium, which blocked warm activated Ca^2+^-permeable Trpv4 (Liedtke et al., 2000), Trpv3 (Tousova et al., 2005) and Trpm2 (Kraft et al., 2004) channels, we found the overall temperature response was largely still present; we observed higher ACf at lower temperatures, however, the effect plateaued after 27°C in that we did not see higher ACf at 22°C. We hypothesize that gadolinium is targeting Trpv4 in these experiments; at temperatures of 22°C, 27°C and 32°C, in the presence of gadolinium we see an increase in ACf compared to control, which fits our Trpv4/SK functional coupling model of PVN neurones where a reduction in Trpv4 *Po* would lead to an increase in ACf. Surprisingly, at 37°C, where we know Trpv4 *Po* would be very high, we did not see any increase in ACf when Trpv4 was inhibited, indicating that another ion channel may be involved.

The remaining target, Trpm2, has been identified as a heat sensor in the POA (Song et al., 2016), is gadolinium insensitive (Harteneck, 2005) and is activated by temperatures >35°C (Togashi et al., 2006). We therefore repeated our temperature procedure in the presence of 10 μM econazole which is known to inhibit Trpm2 currents (Hill et al., 2004) and found that the temperature effect on ACf at all temperatures was blocked, indicating a role for Trpm2 in thermosensing in the PVN. In the presence of econazole, compared to control recordings (no drug), we found that ACf was increased at all temperatures apart from room temperature (22°C) and the effect was most pronounced at 37°C which is just above is the temperature activation threshold of Trpm2.

Interestingly, Song *et al.* (2016) suggested that Trpm2 in preoptic hypothalamic neurones modulates fever responses via the PVN (Song et al., 2016). They found that inhibition of Trpm2^+^ POA neurones resulted in a significant increase in T_c_. This may fit in with our results; we did not patch isolated PVN neurones, but PVN neurones in their somewhat native neuronal environment and thus, any interference from neighboring hypothalamic nuclei such as the POA may be preserved in our brain slice experiments. We show that at 37°C, inhibition of Trpm2 results in a 10-fold increase in ACf, which we hypothesize would lead to an increased sympathetic output and vasoconstriction, which may result in an increase in T_c_. In our single channel electrophysiology experiments, we did not observe a Trpm2-like channel, so we cannot comment on the presence of Trpm2 on PVN neurones or add to the kinetic profile of Trpm2.

We propose that both Trpv4 and Trpm2 are necessary in combination to account for our electrophysiological results; at physiological temperatures we know Trpv4 activity will be high (Figure 3A) and we assume that Trpm2 will be active as we are over the temperature activation threshold. This hypothesis is somewhat dependent on the assumption that Trpv4 properties are similar in our slice preparation to that of recombinant channels in expression systems. Since (as discussed above) some properties of Trpv4 are dependent upon intracellular co-factors, this is not necessarily the case (Watanabe et al., 2002). However, we used exclusively cell-attached patch recording which has the advantage over whole-cell or cell-free patch clamp that the native intracelllular mileu does remain intact. We found inhibiting Trpv4 (with gadolinium) had no effect on ACf, presumably because Trpm2 is still active and conducting enough Ca^2+^ to maintain SK channel activity. This may be surprising considering Trpv4 has a permeability ratio Ca^2+^ to Na^+^ (PCa/Pna) of 6 (Clapham et al., 2003), whereas Trpm2 PCa/PNa is approximately only 0.7 (Sano et al., 2001; Kraft et al., 2004) but we know from our mathematical model that only a small about of Ca^2+^ entry is required to activate nearby K_Ca_ channels. Further work is necessary to confirm the presence of Trpm2 (as opposed to a closely related channel) in the PVN and to determine the comparative expression profile of Trpv4 and Trpv2. As we cool to room temperature, we presume Trpm2 will be switched off first (sub 35°C) whereas Trpv4 will be constitutively active even at room temperature, albeit with low Po (Figure 3A). Inhibiting Trpm2 at room temperature has no significant effect on ACf (presumably as Trpm2 is already switched off), whereas inhibition of Trpv4 results in a significant increase in ACf, suggesting that Trpv4 plays a role in determining neuronal activity at 22°C. We find that blocking Trpm2 with econozole appears to inhibit the temperature effect; ACf is markedly increased (compared to control) at 37°C and remains consistently high as the temperature is lowered. In addition, in our mathematical model, both the presence of Trpv4 and Trpm2 were necessary to account for our physiological data, further suggesting that multiple TRP channels orchestrate the observed response.

In conclusion, our data suggest that cooling temperature challenge inhibits multiple TRP channels including Trpv4 and Trpm2. Our mathematical model predicts that resulting decreases in intracellular Ca^2+^ would inhibit local SK channels, depolarise neurones and hence increase ACf and our experimental patch-clamp data validates this. Together, these data give insight into the important fundamental mechanisms by which the body decodes temperature signals and maintains homeostasis in an area of the brain adapted to control of the cardiovascular control system.

## Data Availability

The original contributions presented in the study are included in the article/Supplementary Material, further inquiries can be directed to the corresponding author.
