# Supplementary figures and images for: Temperature modulates PVN pre-sympathetic neurones via transient receptor potential ion channels
Source: Front Pharmacol. 2023 Oct 18;14:1256924. doi: 10.3389/fphar.2023.1256924 (PMC10618372; doi:10.3389/fphar.2023.1256924)

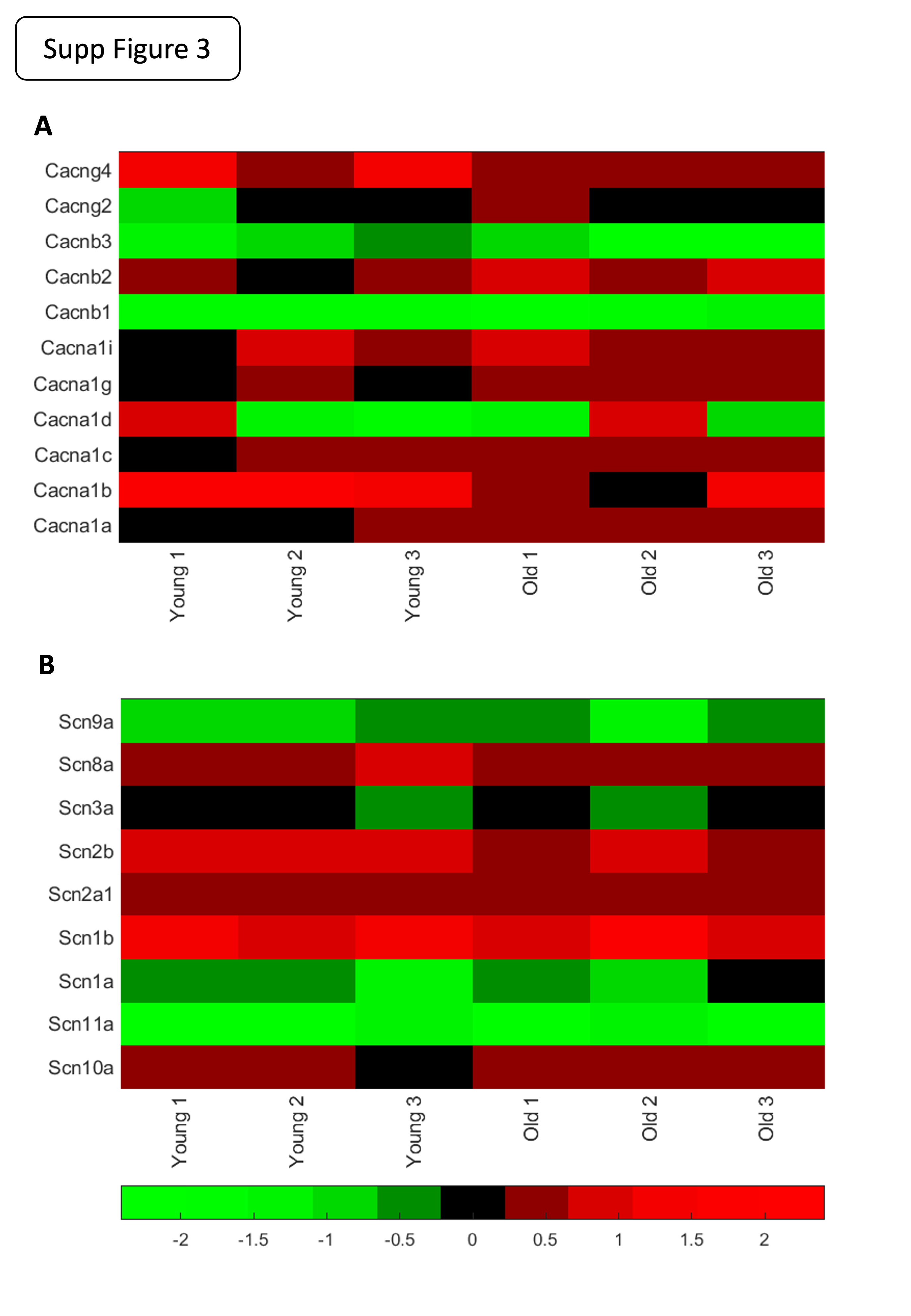

Supplement: Supplementary file 1 [file Image3.TIFF]

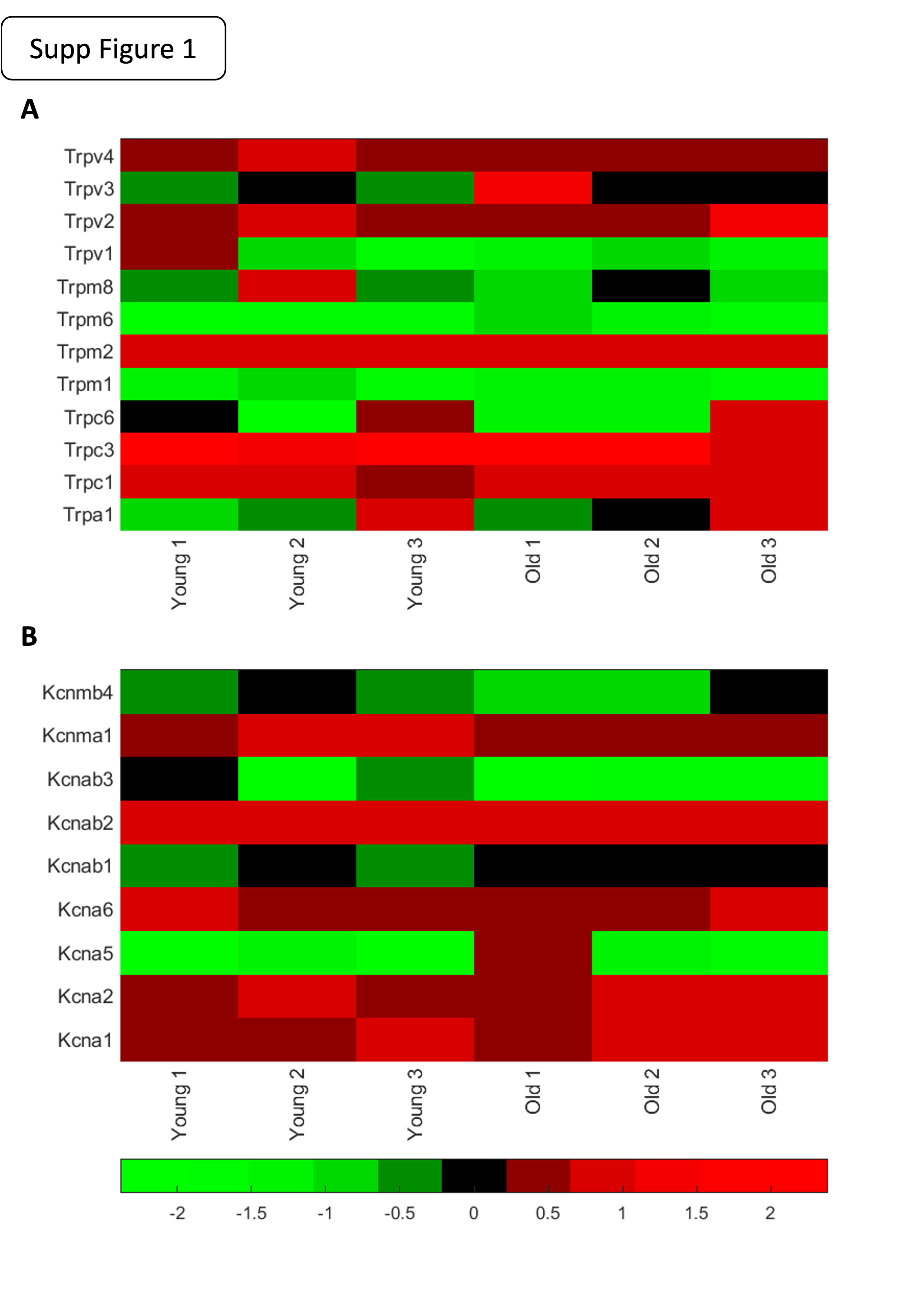

Supplement: Supplementary file 2 [file Image1.TIFF]

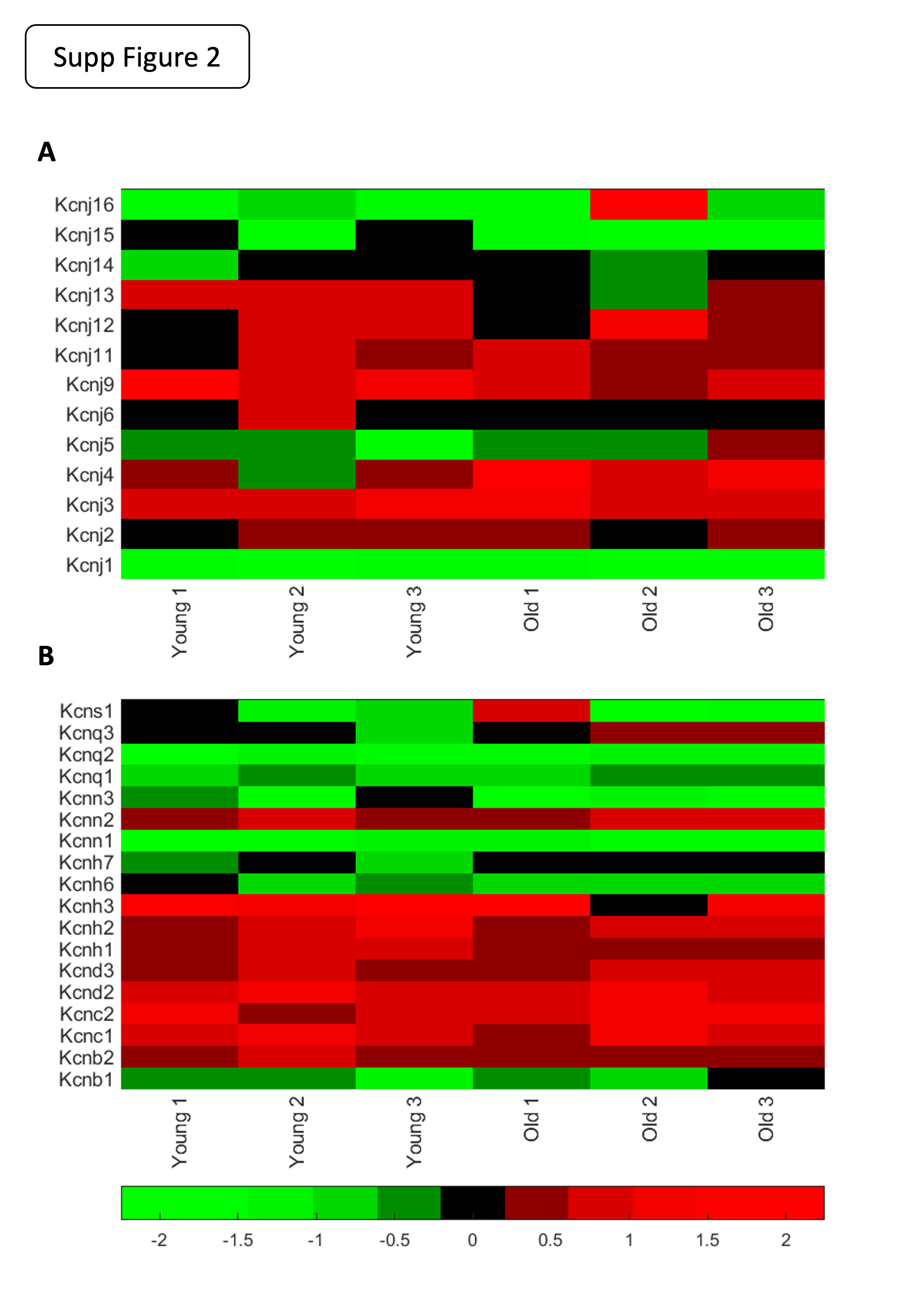

Supplement: Supplementary file 3 [file Image2.TIFF]

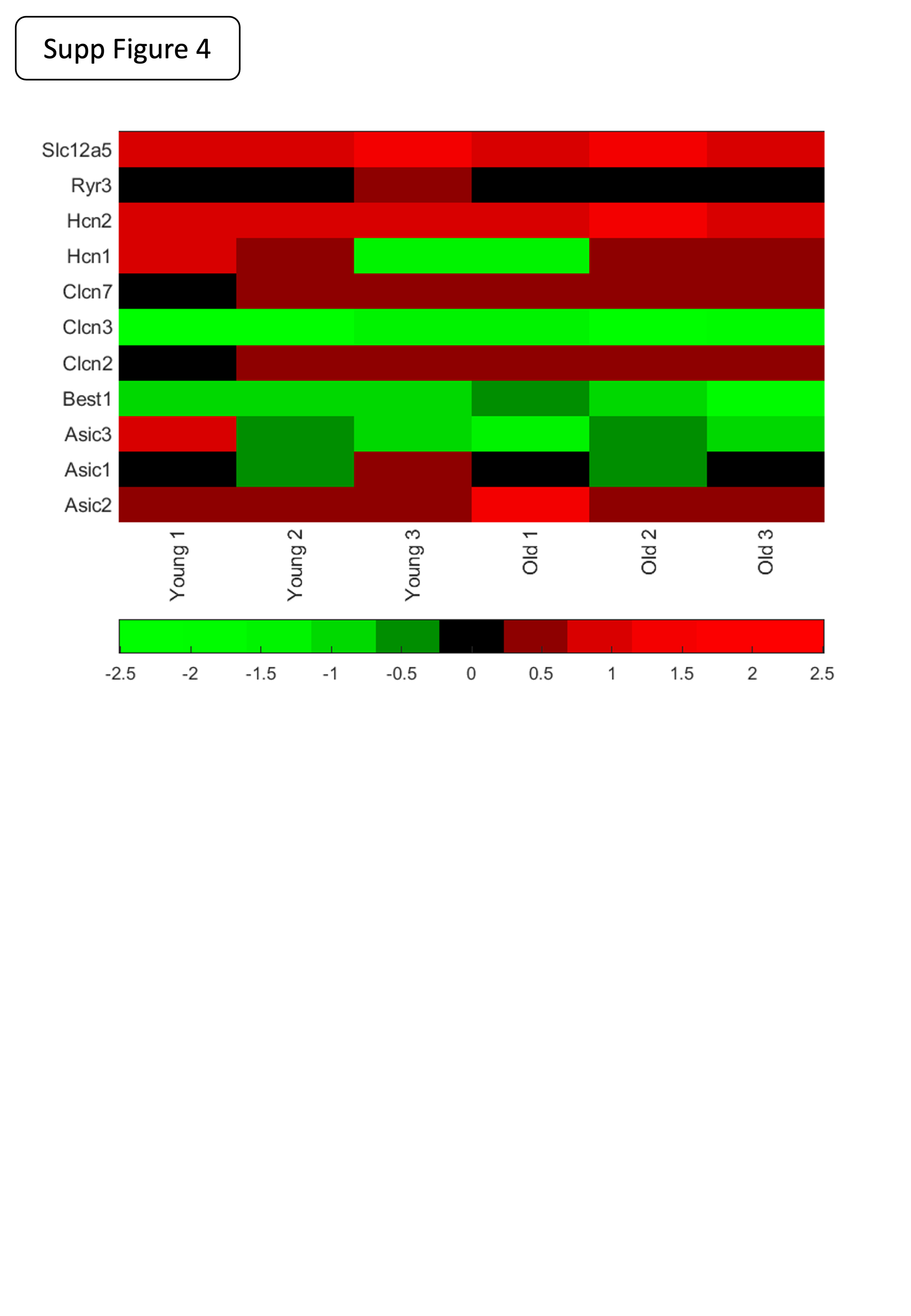

Supplement: Supplementary file 4 [file Image4.TIFF]
