# Supplementary material for: Temperature modulates PVN pre-sympathetic neurones via transient receptor potential ion channels
Source: Front Pharmacol. 2023 Oct 18;14:1256924. doi: 10.3389/fphar.2023.1256924 (PMC10618372; doi:10.3389/fphar.2023.1256924)
Supplement: Supplementary file 5 [file DataSheet1.docx]

**Supplementary figures**

**Supplementary Figure 1. Ion channel gene expression in PVN punches from mice; TRP channels and calcium-activated potassium channels**.

Standardized ΔCt levels for mRNA in punches of the PVN from 3 young adult (6-8 month) and 3 old (26 month) animals. (A) 12 different TRP channel genes. (B) 9 calcium-activated potassium channel related genes. Red genes are relatively highly expressed and the green are low expression. Raw data in Supplementary Table 1.

**Supplementary Figure 2. Ion channel gene expression in PVN punches from young adult and old mice; inwardly rectifying potassium channels and other potassium channels.**

Standardized ΔCt levels for mRNA in punches of the PVN from 3 young adult (6-8 month) and 3 old (26 month) animals. (A) Inwardly rectifying potassium channel genes. (B) Other potassium channel related genes. Red genes are relatively highly expressed and the green are low expression. Raw data in Supplementary Table 1.

**Supplementary Figure 3. Ion channel gene expression in PVN punches from young adult and old mice; calcium ion channels and sodium ion channels.**

Standardized ΔCt levels for mRNA in punches of the PVN from 3 young adult (6-8 month) and 3 old (26 month) animals. (A) Calcium voltage-gated ion channel related genes. (B) Sodium ion channel related genes, not included in the above families together with the transporter Slc12a5. Red genes are relatively highly expressed and the green are low expression. Raw data in Supplementary Table 1.

**Supplementary Figure 4. Ion channel gene expression in PVN punches from young adult and old mice; “other” ion channels.**

Standardized ΔCt levels for mRNA in punches of the PVN from 3 young adult (6-8 month) and 3 old (26 month) animals. Ion channel related genes, not in the previous sets. The colour bar pertains to A and B. Red genes are relatively highly expressed and the green are low expression. Raw data in Supplementary Table 1.

**Supplementary Table 1:**

**:**

Expression relative to mean of 3 housekeeper gene (Actb, Ldha, Rplp1) Cts for that sample is presented as the ΔCt. *n*=3 for each value*.*

| **Gene** | **Young (ΔCt)** | | **Old (ΔCt)** | |
| --- | --- | --- | --- | --- |
| **Asic2** | 3.27±0.21 | 1.75±0.67 | |  |
| **Asic1** | 5.92±1.23 | 7.05±0.19 | |  |
| **Asic3** | 8.46±3.36 | 12.84±2.49 | |  |
| **Best1** | 11.85±0.78 | 12.61±2.19 | |  |
| **Cacna1a** | 4.46±0.65 | 4.39±0.78 | |  |
| **Cacna1b** | -1.38±0.37 | 2.93±2.29 | |  |
| **Cacna1c** | 4.11±0.37 | 4.25±0.82 | |  |
| **Cacna1d** | 8.84±3.22 | 8.5±2.41 | |  |
| **Cacna1g** | 4.66±0.04 | 4.29±0.83 | |  |
| **Cacna1i** | 3.38±0.4 | 3.26±0.59 | |  |
| **Cacnb1** | 13.44±1.13 | 15.17±1 | |  |
| **Cacnb2** | 3.57±0.84 | 2.68±0.89 | |  |
| **Cacnb3** | 8.57±0.84 | 14.87±1.79 | |  |
| **Cacng2** | 5.97±0.55 | 4.93±0.49 | |  |
| **Cacng4** | 0.85±1.3 | 3.6±0.8 | |  |
| **Clcn2** | 4.89±0.08 | 3.99±0.63 | |  |
| **Clcn3** | 18.34±0.85 | 17.13±0.35 | |  |
| **Clcn7** | 4.39±0.15 | 3.39±0.75 | |  |
| **Hcn1** | 6.96±4.22 | 7.95±3.84 | |  |
| **Hcn2** | 0.66±0.33 | -0.05±0.74 | |  |
| **Kcna1** | 1.72±0.23 | 1.09±0.7 | |  |
| **Kcna2** | 2.14±0.49 | 0.95±0.64 | |  |
| **Kcna5** | 15.56±0.69 | 10.24±2.51 | |  |
| **Kcna6** | 1.98±0.56 | 1.67±0.81 | |  |
| **Kcnab1** | 7.08±0.4 | 5.71±0.33 | |  |
| **Kcnab2** | 0.6±0.26 | 0.06±0.55 | |  |
| **Kcnab3** | 10±4.03 | 15.18±0.95 | |  |
| **Kcnb1** | 9.99±0.83 | 8.91±1.32 | |  |
| **Kcnb2** | 4.53±0.08 | 4.57±0.71 | |  |
| **Kcnc1** | 3.09±0.17 | 2.32±0.83 | |  |
| **Kcnc2** | 2.9±0.95 | 1.41±1.03 | |  |
| **Kcnd2** | 2.44±0.11 | 2.14±0.82 | |  |
| **Kcnd3** | 3.91±0.22 | 3.09±0.8 | |  |
| **Kcnh1** | 4.05±0.41 | 4.45±0.6 | |  |
| **Kcnh2** | 3.34±0.91 | 3.31±0.87 | |  |
| **Kcnh3** | -0.24±1.37 | 0.7±2.93 | |  |
| **Kcnh6** | 8.61±1.31 | 11.29±0.49 | |  |
| **Kcnh7** | 9.11±0.84 | 7.3±0.46 | |  |
| **Kcnj1** | 15.88±2.16 | 17.13±0.35 | |  |
| **Kcnj11** | 5.15±0.14 | 4.5±0.66 | |  |
| **Kcnj12** | 3.68±0.64 | 4.41±1.89 | |  |
| **Kcnj13** | 3.34±0.51 | 7.07±1.4 | |  |
| **Kcnj14** | 7.33±0.09 | 7.34±0.81 | |  |
| **Kcnj15** | 10.91±3.7 | 17.13±0.35 | |  |
| **Kcnj16** | 13.63±1.53 | 9.13±4.44 | |  |
| **Kcnj2** | 5.67±0.52 | 5.32±0.64 | |  |
| **Kcnj3** | 2.64±0.19 | 2±0.68 | |  |
| **Kcnj4** | 6.63±1.79 | 0.62±0.85 | |  |
| **Kcnj5** | 10.59±2.23 | 7.13±1.99 | |  |
| **Kcnj6** | 5.51±1.29 | 7.3±0.23 | |  |
| **Kcnj9** | 1.02±1.41 | 2.86±0.82 | |  |
| **Kcnk1** | 16.08±0.6 | 10.71±1.82 | |  |
| **Kcnma1** | 1.56±0.28 | 2.19±0.52 | |  |
| **Kcnmb4** | 6.39±0.49 | 7.62±0.99 | |  |
| **Kcnn1** | 13.67±0.72 | 14.07±0.51 | |  |
| **Kcnn2** | 4.69±0.19 | 3.6±0.83 | |  |
| **Kcnn3** | 9.38±1.99 | 14.62±1.32 | |  |
| **Kcnq1** | 9.6±0.06 | 9.14±0.8 | |  |
| **Kcnq2** | 13.77±0.48 | 13.56±0.96 | |  |
| **Kcnq3** | 7.82±0.98 | 5.4±0.73 | |  |
| **Kcns1** | 10.15±1.55 | 10.99±3.34 | |  |
| **Ryr3** | 5.55±0.03 | 5.97±0.56 | |  |
| **Scn10a** | 3.63±0.24 | 2.85±0.61 | |  |
| **Scn11a** | 11.84±1.18 | 13.92±2.96 | |  |
| **Scn1a** | 6.94±0.62 | 5.7±0.63 | |  |
| **Scn1b** | -0.28±0.25 | -1.06±0.68 | |  |
| **Scn2a1** | 2.29±0.17 | 1.62±0.64 | |  |
| **Scn2b** | 1.39±0.12 | 0.92±0.69 | |  |
| **Scn3a** | 5.11±0.5 | 4.06±0.63 | |  |
| **Scn8a** | 1.79±0.16 | 1.3±0.82 | |  |
| **Scn9a** | 7.24±0.57 | 6.68±0.59 | |  |
| **Slc12a5** | -0.07±0.38 | -0.01±0.57 | |  |
| **Trpa1** | 8.48±2.34 | 8.1±1.86 | |  |
| **Trpc1** | 4.99±0.28 | 4.65±0.25 | |  |
| **Trpc3** | -1.53±1.88 | -1.85±2.09 | |  |
| **Trpc6** | 11.2±3.55 | 12.67±3.41 | |  |
| **Trpm1** | 15.68±0.78 | 17.13±0.35 | |  |
| **Trpm2** | 4.04±0.36 | 3.92±0.67 | |  |
| **Trpm6** | 17.77±0.51 | 16.87±0.36 | |  |
| **Trpm8** | 8.78±1.5 | 12.2±1.38 | |  |
| **Trpv1** | 11.5±2.97 | 15.06±0.98 | |  |
| **Trpv2** | 5.3±0.32 | 4.35±0.86 | |  |
| **Trpv3** | 9.39±0.35 | 6.49±1.64 | |  |
| **Trpv4** | 4.56±0.21 | 7±0.51 | |  |

**Estimation of Resting Membrane Potential Change**

Estimation of resting membrane potential (RMP) changes with temperature; since cell-attached patch single-channel amplitude (*i*) is a function of unitary conductance (*g*)*,* holding potential (*V_h_*)*, channel reversal potential* (*V_rev_*) and *RMP*. Change of RMP (*Δ*RMP) can be calculated as (assuming *V_rev_* , *g* and *V_h_* are constant)

*ΔRMP* = (*i_2_* – *i_1_)* / *g*

Given these assumptions, and the fact that there is an approximately 0.7pA change of single channel amplitude at -50mV, we estimate that RMP could be approximately 10mV less at lower temperatures.
